# Drug-Eluting Stents: Technical and Clinical Progress

**DOI:** 10.3390/biomimetics8010072

**Published:** 2023-02-09

**Authors:** Maciej Koźlik, Jan Harpula, Piotr J. Chuchra, Magdalena Nowak, Wojciech Wojakowski, Paweł Gąsior

**Affiliations:** 1 Division of Cardiology and Structural Heart Disease, Medical University of Silesia, 40-635 Katowice, Poland; 2 Students’ Scientific Society, Department of Cardiology, Faculty of Medical Sciences in Katowice, Medical University of Silesia, 40-635 Katowice, Poland

**Keywords:** drug-eluting stents, scaffold, overexpansion, drug, polymer, coating technique

## Abstract

Drug-eluting stents (DES) demonstrated superior efficacy when compared to bare metal stents and plain-old balloon angioplasty and are nowadays used in almost all percutaneous revascularization procedures. The design of the stent platforms is constantly improving to maximize its efficacy and safety. Constant development of DES includes adoption of new materials used for scaffold production, new design types, improved overexpansion abilities, new polymers coating and, finally, improved antiproliferative agents. Especially nowadays, with the immense number of available DES platforms, it is crucial to understand how different aspects of stents impact the effect of their implantation, as subtle differences between various stent platforms could impact the most important issue—clinical outcomes. This review discusses the current status of coronary stents and the impact of stent material, strut design and coating techniques on cardiovascular outcomes.

## 1. Introduction

The very first percutaneous coronary transluminal angioplasty (PTCA) conducted in 1977 by Andreas Gruentzig [1] started a revolution in the treatment of coronary artery disease. However, initially, the adoption of balloon angioplasty was limited by high rates of restenosis driven mostly by elastic arterial recoil [2]. In order to address this significant limitation, the first coronary bare metal stent (BMS) was developed. This platform was a self-expanding, stainless-steel mesh-like scaffold, hidden in an outer layer sheet, which, when withdrawn, resulted in stent expansion. The first human implantation was performed in 1986 by Ulrich Sigwart. A few years later, Julio Palmaz and Richard Schatz developed the first balloon-expandable platform, which was approved by the FDA in 1994, and since then coronary stents have dominated the field of interventional cardiology. [3] However, despite the fact that they improved acute outcomes by eliminating elastic recoil, the 6-month follow up demonstrated significant rates of neointimal hyperplasia. leading to restenosis observed in up to 30% of cases [4].

The introduction to clinical practice of the first-generation drug-eluting stents (DES) reduced the in-stent restenosis rates to below 6% in selected cases, and as initial randomized controlled trial (RCT) showed, when compared to the BMS, DES reduced the target lesion revascularization (TLR) rates by 74% 1 year after implantation [5,6]. However, the presence of an artificial implant might be associated with impaired vessel-wall healing due to delayed endothelization, and the forming of endothelium with impaired functioning and chronic inflammatory reactions. The mentioned factors promote neoatherosclerosis development, which increases the risk of late in-stent restenosis (ISR), as well as late and very-late stent thrombosis, which were sparse in BMS at the same time follow-up [7,8]. Therefore, prolonged dual antiplatelet therapy for at least 12 months was mandatory for the first-generation DES [9,10]. Constant development of DES and optimized antiproliferative drugs improved vessel healing and the overall safety of coronary stents [11,12]. Moreover, all of the technological upgrades have not reduced mortality or consecutive myocardial infarction rates [13], and repeat revascularization is needed at a rate of 1–2 % per year [14].

Stent coating is also crucial in order to improve device biocompatibility. There are various coating techniques and the most popular are spray coating, dip coating, electrospinning and direct-write inkjet [15]. Furthermore, different types of applied polymers are used nowadays, including durable (DP), biodegradable polymers (BP) as well as polymer-free stents platforms (PF). Biodegradable polymers were developed to reduce inflammatory reactions and lower occurrences of the late and very-late stent thrombosis by eliminating the constant exposure of polymer to vessel healing, which occurs in (DP)-DES stents. However, recent studies comparing clinical outcomes of biodegradable polymer (BP)-DES and durable polymer (DP)-DES showed conflicting results, and some studies failed to demonstrate the superiority of BP-DES.

Therefore, this demonstrates that stent thrombosis is multifactorial and could occur due to every stent component—polymer biocompatibility, strut thickness and design, scaffold material and manufacturing process, as well as antiproliferative drug choice [16]. Similar situations were observed in the BMS, where further development of materials and design improved procedural outcomes. Today’s stents are manufactured with the use of much thinner struts than the initial stainless steel, and have much more advanced platforms design and coating. All of these lowered the risk of restenosis and thrombosis, yet there is need for further improvements not only in stent features but also in pharmacological therapy, to fully overcome these adverse reactions. Consequently, historical strategies of dual antiplatelet therapy are now being challenged by monotherapy using just potent and selective P2Y12 inhibitors.

The various scaffold materials, polymers and antiproliferative agents used nowadays result in an immense number of different platforms. Hereby, we present a review regarding the status of coronary stents with particular focus on their structure, materials, and technological process. The bioresorbable stents, which held great hopes in the recent past, are currently in retreat as large trials, such as ABSORB, have shown an elevated risk of adverse outcomes after implantation, such as TLR, target lesion failure (TLF) or stent thrombosis (ST) [17]. Due to these reasons, they will not be described in this review.

## 2. Stent Design and Outcomes

### 2.1. Stent Scaffold

The scaffold is the “backbone” of the stent. Its key features include the type of metal alloy, thickness of the stent struts and the strut architecture. Each of these features, individually and collectively, influence radial and longitudinal strength, deliverability, conformability as well as fluoroscopic visibility, which might play a crucial role in performing a successful PCI. Easy stent delivery and adequate stent expansion are device-related key factors in initial procedural success. Furthermore, minimizing arterial-wall injury during implantation and facilitating the endothelialization process might play an important role in improving long-term success.

First-generation DES were manufactured using the 316L stainless steel (316L-SS)–material mainly composed of iron, chromium, nickel, molybdenum, and small amounts (<1%) of silicon, sulfur, and phosphorus. The crystalline structure of the 316L-SS makes it relatively durable, corrosion resistant and biocompatible [18]. However, the permanent presence of 316L-SS in arterial walls may trigger an immune response and inflammatory reactions due to the release of metals, with the alloy leading to intimal hyperplasia and in-stent restenosis [18,19]. Furthermore, due to an inherent limitation of 316L-SS, a reduction in strut thickness without sacrificing radial strength is challenging. Therefore, the strut thickness of the first generation ranged between 132 and 140 um. There is also evidence that strut thickness plays an important role in stent restenosis. In the ISAR-STEREO trial, the use of thin-strut instead of thick-strut devices was associated with a significant reduction in restenosis [20,21]. This might be potentially explained by increased local coronary inflammation and greater vessel injury promoting in-stent neointimal growth and hyperplasia, eventually leading to restenosis in thick-strut platforms. In addition, the adverse impact of increased strut thickness on a stent’s surface coverage and healing has been well-described [22]. Therefore, in second-generation DES, stainless steel (SS) ws replaced with chromium-based alloys such as chromium-cobalt (CrCo) or platinum-chromium (PtCr). Two important components of these metal-alloy platforms are the yield strength and elastic modulus. Yield strength, defined as the maximum stress that can be applied to the device before the stent is permanently deformed, directly impacts a stent’s expansion capacity. The elastic modulus is the measure of a stent’s ability to resist deformation when stress is applied. A lower elastic modulus implies less stiffness, leading to improved tracking and conformability of the stent system [23]. Both CrCo and PtCr have higher yield as compared to 316L-SS. Furthermore, it appears that CrCo has a higher elastic modulus compared to PtCr [23]. CrCo is the most widely used material alloy in contemporary DES. In addition, it is chemically inert, which translates into increased biocompatibility by reducing risk of immunological response or irritation [24,25]. Moreover, to improve vessel reaction to artificial stents, the outer surface could be coated with chromium oxide, which acts as a barrier preventing stent ions from reacting with the vessel wall; however, there is no data for the clinical use of this enhancement [26]. Finally, the chromium alloy is denser than steel, which enhances platform visibility under fluoroscopy. PtCr is the second most used material alloy. Platinum itself is chemically stable, corrosion-resistant and biocompatible. Similarly to CoCr, it is denser and stronger than 316L-SS, allowing the adoption of thinner struts and the lower profile of the device, as well as increasing its deliverability to challenging lesions [27]. Furthermore, when compared to the CoCr alloy, the PtCr presents a lower potential of recoil, as the Platinum is more elastic. Furthermore, Resolute Integrity zotarolimus-eluting stents are manufactured from a single 91 μm cobalt alloy wire, which was first wave-shaped (sinusoidal), then helically wrapped and enhanced by local laser fusion. The newest itineration of Resolute stent (Onyx) consists of a single continuous wire, which is sinusoidal-shaped, helically wrapped and locally laser-fused. However, the Resolute Onyx wire is only 81 μm in diameter and consists of two components: a cobalt alloy shell and a platinum-iridium core. Thinner struts promise better deliverability and a platinum-iridium core allows for high radial strength and increased radiopacity, despite a thinner profile. Mentioned features of chromium-based platforms allowed the development of stents with much thinner struts when compared to 316L-SS, without loss of the radial strength. This translated into a thinner profile for the whole device, which improves maneuverability and deliverability to the target lesion. Currently, the cut-off for thick stents is over 100 μm, the thin stents are defined as 70–100 μm, and lower than 70 μm is considered an ultra-thin strut. A smaller surface area accelerates endothelialization and strut coverage after implantation. According to the recent meta-analysis of 16 randomized trials, ultrathin-strut DESs were associated with reduced risks of TLF and target-vessel failure (TVF) compared with conventional second-generation thin-strut DESs [28]. Subgroup analysis according to the different DES types and comparators showed no relative interaction, supporting the concept that the ultra-thin strut design was responsible for the advantage reported in these trials. However, overexpansion of such thin stents could mean losing their mechanical properties [29]. Furthermore, adoption of thin struts was associated with a significant reduction IN excessive neointimal proliferation which, in turn, resulted in a decrease in restenosis rates.

**Figure 1:**
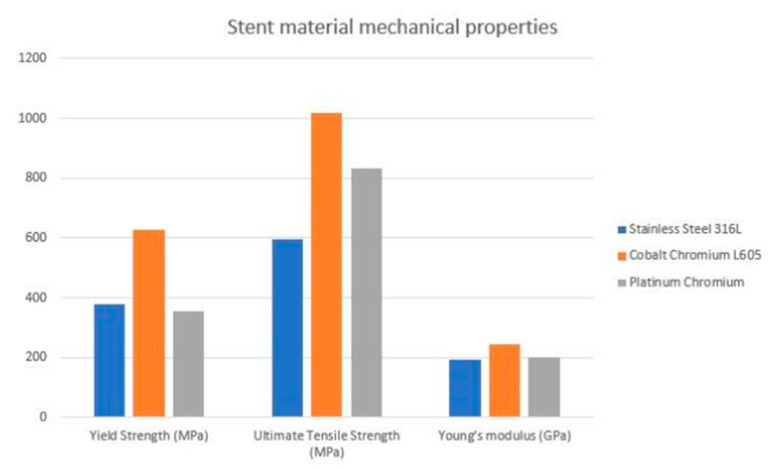
Estimated visualization of mechanical properties of the commonly used alloys.

As the data proves, the cobalt-chromium platforms have been studied in real-life clinical situations. Both CoCr and PtCr stents had better results and outcomes than first-genetation SS stents [30,31,32,33]. The XIENCE V/PROMUS study aimed to examine outcomes with CoCr Everolimus Eluting Stent (EES). At 5-year follow-ups, the major adverse cardiac and cerebrovascular events (MACCE) ratio was 10.7% and clinically driven target vessel revascularization (CD-TLR) was 6.0%, with an annual ratio of 0.5–0.8%—as the authors suggested, these incident ratios are reasonably low, which proves clinical availability [34]. In the HOST-ASSURE study, authors compared outcomes with a CoCr zotarolimus eluting stent (ZES) and PtCr EES—in terms of clinical outcomes, the results’ differences were non-significant in TLF, MACCE and stent post-deployment deformation [30]. However, there is no direct comparison with CrCo platforms which could establish characteristics for the ideal stent design. The estimated visualization of mechanical properties of the alloys most commonly used in clinical practice is presented in a Figure 1 [35].

### 2.2. Stent Cells Design

**Figure 2:**
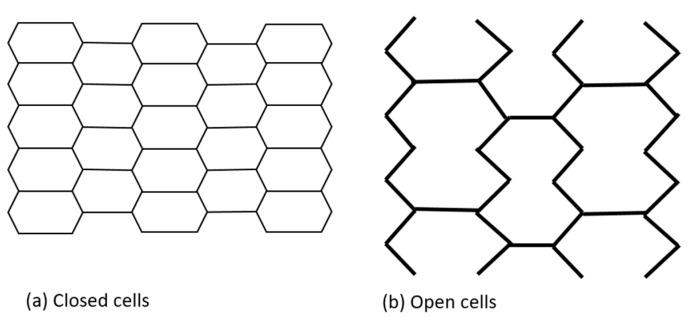
Stent cells geometry.

Initially, coronary stents resembled a coil, which provided flexibility but at the expense of radial strength. They were later replaced by slotted tube stents made from metallic tubes laser cut into desired shapes, which improved radial force but significantly reduced flexibility and deliverability [33,36]. Contemporary platforms have a modular design consisting of repeating rings that form a tube. This design balances the interplay between the need for greater radial force with increased flexibility. Depending on the number of connectors linking each hoop, either an open or closed cell architecture is possible. Furthermore, the number of connectors determines the deliverability, flexibility, and conformability of the stent. The area between two consecutive rings and their connectors is defined as a stent cell. A closed cell design is characterized by a high number of connectors. In turn, an open cell design is defined by lower numbers of connectors and larger stent cells. A closed cell design is associated with increased radial strength, higher wall coverage and potentially less plaque prolapse, while an open cell design improves deliverability, flexibility and conformability while reducing side branch compromise. Even though an open cell design is associated with potentially lower radial strength, it is used in all of the new-generation platforms, which usually have only two to three connectors (Figure 2). The selected stents, with their characteristics, are presented in Table 1.

### 2.3. Bifurcation Design

With increasing operator experience and improved devices, there is an increase in the number of complex PCI, including bifurcation lesions. These pose challenges for optimal clinical effect, as those procedures are associated with an increased number of periprocedural complications as well as thrombosis and restenosis rates. Nowadays, bifurcation interventions constitute up to 20% of all PCI procedures [48]. Key factors for successful bifurcation PCI include appropriate stent sizing, correct positioning with the help of intravascular imaging and adoption of an appropriate technique. However, achieving optimal stent expansion in the proximal part, which is wider, without the risk of dissection in the distal part of the main branch, is often challenging. Furthermore, securing the side branch (SB) ostia is crucial—with inadequate technique there is a chance of shifting the carina towards SB as well as shifting the plaque towards carina, which reduces the SB ostia lumen area. The occlusion of SB is considered as one of the most common complications during bifurcation interventions, simultaneously making these procedures complex and challenging [49,50]. There are a number of different approaches to bifurcation lesions, such as: provisional stenting (PS), double kissing crush, T-stenting and Culotte [51]. Stents used for bifurcations should have significant overexpansion capabilities and appropriately wide ostia in order to provide access to the side branch [52,53]. Besides adequate technique, there are also a few stent platforms specifically designed for bifurcations, such as BIOSS LIM C (Balton, Warsaw, Poland). The BiOSS LIM C^®^ stent is composed of two parts with different diameters: the proximal part and distal part. The proximal part is always shorter than the distal part (1 mm on average) and wider than the distal part. The ratio of proximal/distal parts varies between 1.15 and 1.3. In the midzone, the two parts are connected by two struts (2.0–2.4 mm in length after BiOSS^®^ stent implantation). In the study regarding the use of this platform in real-world clinical situations, after 3-months follow-up, there were 6.3% of MACE, 4.2% of myocardial infarction and 2.1% TLR [54]. Several other platforms were developed specifically for bifurcation settings, including the self-expanding nitinol (nickel and titanium alloy) platform Stentys (Stentys S.A, Paris, France). It was designed for arteries with varying proximal and distal diameters. The mechanical properties of nitinol allow for constant expansion over time after being implanted, which aims for high conformability to the vessel wall. Moreover, it has interconnectors between struts which can disconnect, which could be opened with balloon inflation in order to gain access to the side branch (SB). With the available data, the Stentys was used in clinical practice for patients with both acute coronary syndrome (ACS) and elective PCI—the observed TVR ratio was 7.8%. There was not, however, any head-to-head comparison with classic bifurcation stenting techniques [55]. The planned APPOSITION V trial, regarding Stentys in ACS with ST segment limitation, was terminated after 2 years and no results were presented. Another platform designated for bifurcation is a Tryton (Tryton Medical Inc., Durnham, NC, USA), a Co-Cr platform with a short and wide proximal part for the main branch, flexible transition zone and narrower side branch part. As the manufacturer describes, the Tryton allows integration with other stents implanted. However, in the TRYTON randomized clinical trial, it failed to demonstrate a superiority over provisional bifurcation stenting technique [56]. Despite such advancements in the field of different platforms, bifurcation is still considered one of the most challenging PCI procedures.

### 2.4. Overexpansion Abilities

**Figure 3:**
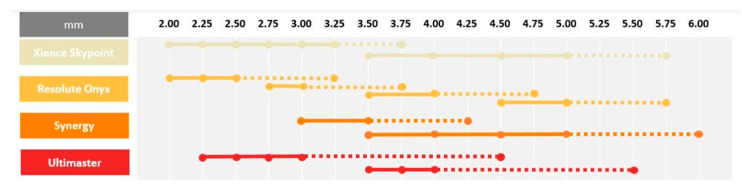
Overexpansion capabilities.

Contemporary DESs are commonly used in daily clinical practice for interventions in left-main and long tapered segments. In both these settings, large discrepancies between the proximal and distal part of the targeted lesion frequently occurs. In this case, the optimal device size must be selected according to the distal diameter of the treated segment, which results in a mismatch between stent size and the proximal diameter of the vessel, which requires post dilatation using larger balloons to ensure optimal strut apposition. In addition, stents are rarely able to achieve a lumen diameter equal to the balloon diameter used for post dilatation [53]. Incomplete stent apposition might be associated with increased rates of adverse events such as in-stent restenosis and stent thrombosis [57]. Contemporary DES platforms have the ability to overexpand way beyond their nominal diameter without risk of strut fracture [58]. However, it needs to be stressed that, despite the fact that these DESs can be oversized without damaging the struts, it does not imply it is completely safe to do so. Extensive overexpansion leading to approaching the physical limits of the stent might lead to the modification of mechanical stiffness and impact the drug delivery process, which, in turn, might completely alter the device performance. An increased risk of polymer-coating damage or the detachment of debris might be associated with a higher prevalence of thrombosis and inflammation, with neointimal reactions [59]. Furthermore, it should be noted that stent overexpansion is associated with a large increase in crown angle. When crown angle exceeds 150°, the stent crowns are almost completely straightened which indicates that the device is reaching its physical limit [53]. While crown straightening results in increased radial force, it also significantly increases stiffness, which can reduce the device durability and make it more prone to fatigue and stent fracture. Moreover, during overexpansion, the cell opening diameter becomes larger, which increases the risk of underlying plaque prolapse in between the struts. In the overexpanded regions, the increased cell diameter can cause a reduction in drug elution per mm^2^, which can lead to a higher risk of excessive neointimal proliferation. Insights from bench tests provide important information which may be helpful for the careful selection of stent size for contemporary DES based on model designs [59]. Such information is especially critical in left-main bifurcation stenosis treatment, where overexpansion to larger, oversized diameters may be required to ensure full stent apposition. Stents with the greatest overexpansion capabilities [60,61,62,63,64] are presented in Figure 3.

## 3. Polymers and Coating

Polymer stent coating is crucial in the controlled release of the antiproliferative agent. This is of utmost importance, since currently used drugs have relatively short half-lives and without an optimal elution profile from the stent surface, they would be unable to effectively inhibit neointimal proliferation over the desired time period. Therefore, in order to be effective, antiproliferative agents are released from the polymer surface, which controls elution kinetics and provides sustained drug delivery during the initial 10–30 days following stent implantation, which is essential to prevent restenosis [65]. Characteristics of a theoretically ideal polymer include high biocompatibility, being biologically inert, a lack of interaction with the antiproliferative drug, durability and resistance to elongation to avoid damage when excessive forces are applied.

**Figure 4:**
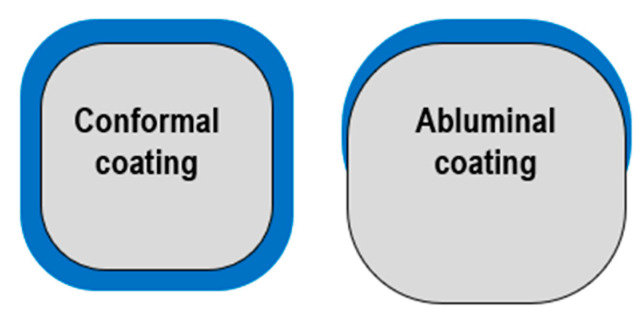
Polymer distribution: conformal vs. abluminal.

However, polymer presence may cause local hypersensitivity reactions, delaying the healing and endothelization process [66] as well as trigger inflammation or neoatherosclerosis, increasing the risk of thrombosis [67,68]. Some studies support the thesis that polymers which are mounted on the stent’s abluminal part can cause less tissue interaction than circumferential polymers. This is due to the drug being directly in contact with the vessel wall and not with plasma in the arteries [66] (Figure 4).

Furthermore, many coating techniques are currently being used in the manufacturing process, each offering its own pros and cons that must be considered when choosing between them. The main coating techniques used in DES are spray coating, electrospinning, the dip-coating technique and direct-write injket. In the spray-coating technique, a nozzle is used to spray solution-forming droplets with the polymer (and a drug) on a rotated setting [15,69]. In the dip-coating technique, the stent is immersed several times into a polymer–drug solution. Thus, after a couple of repeats, a suitable amount of drug is deposed. The advantages of this technique are simplicity and its low cost and it is extensively used for research purposes [15,70]. Nonetheless, there are some disadvantages of the mentioned techniques, which include a loss of waste solution, low coating rate, and the time-consuming process. On the contrary, the direct-write inkjet technique exhibits several advantages, including flexibility, low cost, and high coating yields by controlling sample aspiration from the dispenser nozzle, which is far superior to the spray-coating and dip-coating methods. The direct-write inkjet technique is based on the printing of drug-loaded polymer particles directly onto the metal surface. In addition, with this technique, the droplet size of the polymer–drug can be tuned very precisely. It simultaneously patterns multiple analytes while using independent cartridges, decreases the pollution of the coated material an promotes the release profiles of drugs [15,55,56]. Electrospinning uses an applied voltage to atomize liquid particles. Highly charged small atomized droplets are monodispersed, which leads to highly efficient polymer deposition onto substrates. This technique is very unique and can even offer polymeric scaffolds with similar mechanical properties, such as native vascular tissue [15].

### 3.1. Durable Polymers

The first-generation DES stents used synthetic polymers: poly(ethylene-co-vinyl acetate)—(PEVA), poly(n-butyl methacrylate)—(PBMA) or tri-block copolymer poly(styrene-b-isobutylene-b-styrene)—(SIBS) and were used in Cypher and Taxus Express stents, which are presented in Table 2. However, these polymers were considered to be responsible for chronic hypersensitivity of the vessel wall that potentially delayed the endothelizalization process of the stent struts and subsequently increased the risk of stent thrombosis (ST) [67]. In order to overcome these limitations, more biocompatible polymers with reduced potential for inflammatory response and delayed arterial healing were developed [67,71]. For both the Xience everolimus-eluting stent and PROMUS everolimus-eluting stent, the polymer is made of two layers: a base layer of poly(n-butyl methacrylate)—(PBMA) covered with a fluorinated polymer, poly(vinylidene fluoride-co-hexafluoropropylene)—(PVDF-HFP). Albumins adhere to the fluoropolymers’ surface and, thus, decrease platelet adhesion and activation and other prothrombotic proteins (for instance, fibrinogen) [68]. The Resolute zotarolimus-eluting stent uses a multi-component BioLinx polymer which consists of three elements: a hydrophobic C10 polymer, hydrophilic polyvinylpyrrolidone polymers and both a hydrophilic and hydrophobic polyvinylpyrrolidone C19 polymer. Proteins adhere to hydrophobic BioLinx components and form a hydrophilic layer, which results in the amphiphilic character of the whole polymer surface and is believed to decrease the adhesive ability of other plasma proteins [68].

### 3.2. Bioresorbable Polymers and Polymer-Free Stents

The permanent presence of even improved, more biocompatible polymers have the potential to cause a local inflammatory response. Bioresorbable polymers were developed as a promising strategy to improve biocompatibility and facilitate the endothelization process. A bioresorbable polymer dissolves after the desired period, leaving a residual metallic scaffold similar to BMS, which reduces the potential of polymer interactions with arterial tissue [45,46]. In addition, some polymers are applied solely on the abluminal side of the stent, lowering the chance of interaction between plasma and polymer molecules and, therefore, are believed to reduce the risk of potential adverse events even further by minimizing the polymer burden and reducing drug concentrations, making them, in theory, more biocompatible than a conventional DES; however, this is yet to be proven in large clinical trials [47,72]. Both BP-DES and polymer-free (PF-DES) stents should theoretically decrease or completely eliminate the number of polymer-related adverse events. The vast majority of BP-DES polymers are made of poly(L-lactic acid)—(PLLA) and poly(D,L-lactide)—(PDLLA) [67,68], [poly(lactide-co-glycolide) (PLGA) and polycaprolactone (PCL) or poly(D,L-lactide-*co*-caprolactone (PLCL) [67]. The three first polymers are degraded to lactic acid, and the last one into carbon dioxide and water. BP-DES stents demonstrated even lower ability to cause inflammation compared with DP-DES stents [67]. Such polymers are often thin (for instance PLLA 7.5 µm—abluminal stent surface and 3.5 µm—luminal surface in Orsiro SES), applied solely to the abluminal surface and have a shorter drug-eluting time, which enhances a rapid strut endothelialization process [72,73,74,75,76]. Nevertheless, it is still not certain whether BP are associated with improved outcomes when compared to DP stents. While several studies have not shown the superiority of BP-DES over DP-DES stents [66,68,77], the meta-analysis of randomized trials showed that after 5 years, the frequency of major acute cardiac events (MACE) or TLR was lower for BP-DES stents in comparison with DP-DES stents [19]. The most widely used BP-DES stents are presented in Table 2.

In polymer-free (PF)-DES stents, the drug is directly bonded to the stent’s scaffold and there is no need for any coating or polymers [68]. Today’s technology allows to produce porous nanostructures on stents with metal scaffolds or simply coating the surface (usually the abluminal part), creating a thin film structure with specifically dedicated materials such as drugs. There are various possible structures and surfaces (such as nanotubus, nanoleaves, nanograss, nanoflakes, nanopillars or nanowires and more) that can be fabricated on metallic surfaces in order to elute a drug in a controlled way. Among the various surface types, a specific titania nanoleaf structure showed higher cytocompatibility and haemocompatibility in vitro, with high endothelialization and low smooth-muscle-cell (SMC) proliferation [67]. The production process varies between different companies and the exact description of each separate stent is not the aim of this review. Different surface properties, such as topography, chemistry or roughness, are characterized by different influences on the plasma, proteins and cells, which can become crucial in future studies. The porous surface of a nanotexture can be a space for a drug to be placed, while hydrophilic/hydrophobic nanostructures can regulate the drug-release kinetics. The critical feature in PF-stents is the elution profile of an antiproliferative drug with a shorter elution time (for instance, 100% of a drug is eluted in 1 month in BioFreedom^®^ (Singapore)) [78]. Research showed that platelet cells fail to adhere to the micro/nanostructure [67]. Technologically modified stent surfaces can mimic the natural extracellular matrix and, thus, help to regulate vascular cell adherence and proliferation [45]. Stents without the polymer eliminate the problem of polymer interaction with living tissue, especially reducing the risk of inflammation, delayed endothelial healing or restenosis rate [67,78]. Up-to-date studies have not shown the superiority of PF-DES over other types of stents yet. Some large studies showed that PF-DES stents are associated with higher MACE, TLR, TVR and in-stent late lumen loss than BP-DES stents [79,80]. Furthermore, two large meta-analysis studies demonstrated that there is no difference in myocardial infarction (MI), cardiac death (CD), all-cause death, ST, TLR, TVR and diameter stenosis (DS) between DP-DES and PF-DES [81,82]. However, future development in the field of coronary stents will aim to improve results, improving the efficacy and safety of manufactured stents, including PF-DES. The most widely used worldwide PF-DES are summarized in Table 2.

### 3.3. Eluted Drugs

The primary goal for an eluted drug is to evoke an anti-proliferative response and, therefore, lower the risk of in-stent restenosis (ISR). The first-generation DES introduced paclitaxel and sirolimus. Paclitaxel had a weaker anti-proliferation effect on cells in hypoxia circumstances (vascular stenosis is associated with hypoxia areas) than sirolimus and needed a dose increase in order to work similarly, which can increase toxicity as well [83]. When compared with particular drugs from the -limus family, paclitaxel appeared to have higher TLR rates [84]. The drug mechanism of action concerns the mTOR signaling pathway. When the mTOR receptor is inhibited, it causes the cell cycle to stop in the late G1 phase or S phase and, thus, hinder SMC proliferation. Another mechanism is a displacement of the FKBP12-binding protein, which leads to endothelial cell-barrier impairment. The whole -limus family supports poor endothelial-barrier functioning independently of the mTOR signaling pathway, which partially explains why neoatherosclerosis occurs more rapidly with DES than with BMS [68]. In two meta-analyses, sirolimus reduced the risk of reintervention and ST when compared with paclitaxel [85,86]. Second-generation DP-DES stents use mostly -limus analogues in combination with more biocompatible polymers, which translates into a lower risk of stent-related adverse events when compared to BMS and first-generation DES [71]. Furthermore, new-generation -limus analogues (zotarolimus, everolimus and biolimus) are more lipophilic than sirolimus, which improves coronary-vessel drug uptake. Therefore, they require lower drug doses, which leads to lower cytotoxicity and potentially improved endothelialization processes [68,87]. For years, everolimus was considered as gold standard in the -limus family. Some studies comparing first- and second-generation DES stents have showed lower rates of stent thrombosis in everolimus stents, in comparison with sirolimus or zotarolimus stents (but not the zotarolimus-eluting Resolute stent). What is more, they showed lower rates of TVR and TLR in stents with everolimus, in comparison with sirolimus or zotarolimus stents (but not the zotarolimus-eluting Resolute stent) [84]. Biolimus is most widely used in BP-DES and PF-DES stents and, unlike other -limus analogues, it was specifically developed for coronary-stent usage [87]. In a comparison study of various zotarolimus, everolimus and biolimus stents, there were no significant differences in long-term MACE, all-cause mortality, any revascularization, rehospitalization and stent thrombosis [88]. As previously mentioned, the summary efficacy of a stent is based not only on the drug itself but the entire stent architecture.

### 3.4. Drug Elution Time

The final issue related to the stent coating is the drug elution kinetics. Different platforms are characterized by various elution times. Furthermore, the most optimal timing for drug elution and polymer degradation is still unknown. Theoretically, more rapid antiproliferative-agent elution may allow for accelerated and more complete healing to occur within the first several months after stent implantation, a critical period for vascular restoration to prevent later complications. However, Endeavor Sprint was the DES with one of the most rapid elution times (14 days); however, it showed an increased risk of restenosis when compared to the slow-releasing Resolute DES (180 days); thus, it was withdrawn from the market [89]. Therefore, it is possible that slower drug release is more favorable than rapid elution—this could be due to the use of less toxic doses of antiproliferative drugs, as well as a consistant dose of this medication to the vessel [90]. This was confirmed by human autopsy analyses in which improvements in new polymers’ conformity with the biologic milieu were thought to decrease inflammation and fasten vessel healing and re-endothelization. However, the largest clinical study comparing stents with short and long drug eluting time (PANDA III trial) demonstrated that the rapid-eluting drug BuMA SES (30 days elution) was not inferior to the Excel SES (180 days elution) and had a lower incident ratio for stent thrombosis [91]. Once more, there is no simple answer to which type of drug eluting stent is the best for clinical outcomes. The drug elution time in selected stent platforms is summarized in Table 2.

## 4. Future Directions

Currently, improved modern DES platforms and experienced operators determine relatively low rates of in-stent restenosis and thrombosis. However, constant technological development may further improve the safety and efficacy of the PCI. Such initiatives could include nanoscale engineering [67]. This is rather a new approach in stent manufacturing—nanothin coatings, nanotextured surface, nanofibrous and nanoparticulate stent coatings. Such technology is being tested in in-vitro environments, yet there are very few clinical trials. Nevertheless, the potential benefits of nanomaterials could improve stent-surface modification technology [92]. Furthermore, the current -limus-based analogues work by inhibiting the mTOR with use of the FKBP12 complex, therefore impairing endothelial barrier function. A role for specific mTOR inhibitors, such as ATP-competitive mTOR inhibitors, are potential therapeutic options for local elution in DES, which do not bind FKBP12 for mTOR inhibition. The use of newer, more experimental agents, which selectively target vascular smooth muscle cells, may improve vascular responses to DES and enhance long-term outcomes in patients with CAD [92]. A different approach that may improve with PCI outcomes is the further development of polymer-free platforms, where the drug is eluted throughout pores in the stent scaffold, which accelerates drug eluting and, therefore, reduces the necessary DAPT time, especially in high-bleeding-risk populations [93].

## 5. Conclusions

As demonstrated in this review, coronary stents are very complex devices. The evolution of the stent role in clinical practice required a significant shift from its original design. This was primarily driven by the requirements of the increasingly complex lesions and challenging patients being treated. Each individual component of DES design has an impact on device performance. New scaffolding materials allowed a reduction in strut thickness which facilitated vessel’s short-term healing response while improvements in stent design provided greater overexpansion capabilities, making DES adoption possible even in very complex anatomies. The introduction of new, more biocompatible durable polymers as well as biodegradable polymers in the new-generation DES reduced the number of polymer-related adverse events, which were significantly limiting the performance of the first-generation devices. Furthermore, the enhanced delivery of anti-proliferative agents, and the sustained inhibition of the excessive neointimal proliferation, significantly reduced rates of restenosis. It needs to be stressed that a reduction in restenosis, recurrent myocardial infarction, and stent thrombosis rates in the current-generation DES is based not on its individual component but the entire device design. Furthermore, as the stent design is crucial for acute and long-term cardiovascular outcomes, it is of outmost importance that interventional cardiologists have a complete understanding of the design features of the available devices.

## Figures and Tables

**Table 1:** Difference in stent-platform and drug-carrier specifications.

Type.	Stent Platform Name (Manufacturer)	Stent Platform Material	Strut Thickness (µm)	Polymer/Coating	Polymer Thickness (µm)
DES 1st	Cypher (Cordis) [37,38]	SS	140	PEVA, PBMA	12.6
	Taxus (Boston Scientific) [37,38]	SS	132	SIBS	16
DES 2nd	Xience V (Abbot) [37,38]	Co-Cr	81	PVDF-HFP, PBMA	7.6
	Endeavor Sprint (Medtronic) [37,38]	Co-Cr	91	Phosphorylcholine	4.1
	Resolute (Medtronic) [38,39]	Co-Cr	91	Biolinx polymer	4.1
	Promus (Boston Scientific) [38,39]	Pt-Cr	81	PVDF-HFP	6
	Resolute Onyx DES (Medtronic) [40,41]	Shell: Co Core: Pt-Ir	81	Biolinx polymer	5.6
BP-DES 3rd	Synergy (Boston Scientific) [39,42]	Pt-Cr	74	PLGA	4
	Orsiro (Biotronik) [42,43]	Co-Cr	61	PROBIO, PLLA	7.4
	Ultimaster (Terumo) [42,44]	Co-Cr	80	PLCL	<15
	Biomatrix (Biosensors) [39]	SS	112	PA	10
	Alex Plus (Balton) [45]	Co-Cr	70	PLGA	<10
Polymer-free 3rd	BioFreedom (Biosensors) [39,42,46]	SS	112	No, microabrasion	-
	CRE 8 EVO (Alvimedica) [39,42,47]	Co-Cr	70–80	No	-

SS = stainless steel; Co-Cr = Cobalt chromium; Pt-Cr = Platinum chromium; Pt-Ir = Platinum iridium; PEVA = poly(ethylene-co-vinyl acetate); PBMA = poly(n-butyl methacrylate); SIBS = poly(styrene-b-isobutylene-b-styrene); PVDF-HFP = poly(vinylidene fluoride-co-hexafluoropropylene); Biolinx polymer = hydrophobic C10 polymer and hydrophilic C19 polymer, and PVP (polyvinyl pyrrolidone); PLGA = poly(D,L-lactide-co-glycolide); PLLA = poly(L-lactide); PLCL = poly(D,L-lactide-co-caprolactone); PA = polylacticacid; BRS—Bioresorbable Scaffolds.

**Table 2:** Durable and bioresorbable stent comparison.

Durable/Bioresorbable	Stent (Manufacturer)	Drug (Dosage)	Drug Release (%) Time	Polymer/ Coating	Polymer Biodegradation (months)	Polymer Distribution
Durable	Xience V (Abbot) [37]	Everolimus (1 μg/mm^2^)	80% 340 days 100% 4 months	PVDH-HFP, PBMA	N/A	Conformal
	Endeavor Sprint (Medtronic) [37]	Zotarolimus (10 μg/mm^2^)	95% 14 days	Phospho- Rylcholine	N/A	Conformal
	Resolute Family (Medtronic) [38,39]	Zotarolimus (10 μg/mm^2^)	85% 60 days 100% 6 months	Biolinx polymer	N/A	Conformal
	Promus Family (Boston Scientific) [38,39]	Everolimus (1 μg/mm^2^)	80% 30 days 87% 90 days	PVDF-HPF	N/A	Conformal
	Xience Family (Abbot) [70]	Everolimus (1 μg/mm^2^)	100% 4 months	PBMA, PVDF-HPF	N/A	Conformal
Bioresorbable	Synergy (Boston Scientific) [39]	Everolimus (LD-56 μg/20 mm^2^ SD-113 μg/20 mm^2^)	50% 60 days 100% 120 days	PLGA	3	Abluminal
	Ultimaster (Terumo) [71]	Sirolimus A9 (3.9 μg/mm^2^)	100% 3–4 months	PLCL	3–4	Abluminal
	BioMatrix (Biosensors) [39]	Biolimus A9 (15.6 μg/mm^2^)	45% 30 days	PA	6–9	Abluminal
	Orsiro (Biotronik) [38,43]	Sirolimus (1.4 μg/mm^2^)	50% 30 days 80% 3 months	PROBIO PLLA	>12	Conformal
	Alex (Balton) [45]	Sirolimus (1.0 μg/mm^2^)	100% 8 weeks	PLGA	2	Conformal
Polymer free	Cre 8 (Alvimedica) [39,42,47]	Sirolimus (0.9 μg/mm^2^)	100% 3 months	N/A	N/A	N/A
	BioFreedom (Biosensors) [39,42,46]	Biolimus (15.6 μg/mm^2)^	100% 1 month	N/A	N/A	N/A

N/A = not applicable, PVDF-HFP = poly(vinylidene fluoride-co-hexafluoropropylene); PBMA = poly(n-butyl methacrylate); Biolinx polymer = hydrophobic C10 polymer and hydrophilic C19 polymer, and PVP (polyvinyl pyrrolidone); PLGA = poly(D,L-lactide-co-glycolide); PLCL = poly(D,L-lactide-co-caprolactone); PA = polylacticacid; PLLA = poly(L-lactide).

## Data Availability

Data available in a publicly accessible repository.

## References

[1] Grüntzig A. (1978). Transluminal Dilatation of Coronary-Artery Stenosis. Lancet.

[2] Grüntzig A.R., Senning Å., Siegenthaler W.E. (1979). Nonoperative Dilatation of Coronary-Artery Stenosis. N. Engl. J. Med..

[3] Carrié D., Elbaz M., Andrieu M., Cantié P., Fourcade J., Puel J. (2000). Ten-Year Clinical and Angiographic Follow-up of Coronary Wallstent. Am. J. Cardiol..

[4] Serruys P.W., Strauss B.H., Beatt K.J., Bertrand M.E., Puel J., Rickards A.F., Meier B., Goy J.-J., Vogt P., Kappenberger L. (1991). Angiographic Follow-up after Placement of a Self-Expanding Coronary-Artery Stent. N. Engl. J. Med..

[5] Moliterno D.J. (2005). Healing Achilles—Sirolimus versus Paclitaxel. N. Engl. J. Med..

[6] HILL R. (2004). Drug-Eluting Stents: An Early Systematic Review to Inform Policy. Eur. Heart J..

[7] Nusca A., Viscusi M.M., Piccirillo F., De Filippis A., Nenna A., Spadaccio C., Nappi F., Chello C., Mangiacapra F., Grigioni F. (2022). In Stent Neo-Atherosclerosis: Pathophysiology, Clinical Implications, Prevention, and Therapeutic Approaches. Life.

[8] Komiyama H. (2015). Neoatherosclerosis: Coronary Stents Seal Atherosclerotic Lesions but Result in Making a New Problem of Atherosclerosis. World J. Cardiol..

[9] Sahebjalal M., Curzen N. (2015). Twelve Months Dual Antiplatelet Therapy after Drug-Eluting Stents—Too Long, Too Short or Just Right?. Interv. Cardiol. Rev..

[10] Becker R.C., Helmy T. (2015). Are at Least 12 Months of Dual Antiplatelet Therapy Needed for All Patients With Drug-Eluting Stents?. Circulation.

[11] Madhavan M.V., Kirtane A.J., Redfors B., Généreux P., Ben-Yehuda O., Palmerini T., Benedetto U., Biondi-Zoccai G., Smits P.C., von Birgelen C. (2020). Stent-Related Adverse Events &gt;1 Year after Percutaneous Coronary Intervention. J. Am. Coll. Cardiol..

[12] Moussa I.D., Mohananey D., Saucedo J., Stone G.W., Yeh R.W., Kennedy K.F., Waksman R., Teirstein P., Moses J.W., Simonton C. (2020). Trends and Outcomes of Restenosis After Coronary Stent Implantation in the United States. J. Am. Coll. Cardiol..

[13] Kheiri B., Osman M., Abdalla A., Ahmed S., Bachuwa G., Hassan M. (2018). The Short- and Long-Term Outcomes of Percutaneous Intervention with Drug-Eluting Stent vs Bare-Metal Stent in Saphenous Vein Graft Disease: An Updated Meta-Analysis of All Randomized Clinical Trials. Clin. Cardiol..

[14] Giustino G., Colombo A., Camaj A., Yasumura K., Mehran R., Stone G.W., Kini A., Sharma S.K. (2022). Coronary In-Stent Restenosis. J. Am. Coll. Cardiol..

[15] Yanqin F., Xiang L., Ruijie Y. (2018). The Surface Modifications Methods for Constructing Polymer-Coated Stents. Int. J. Polym. Sci..

[16] Lu P., Lu S., Li Y., Deng M., Wang Z., Mao X. (2017). A Comparison of the Main Outcomes from BP-BES and DP-DES at Five Years of Follow-up: A Systematic Review and Meta-Analysis. Sci. Rep..

[17] Gallinoro E., Almendarez M., Alvarez-Velasco R., Barbato E., Avanzas P. (2022). Bioresorbable Stents: Is the Game Over?. Int. J. Cardiol..

[18] Fu J., Su Y., Qin Y.-X., Zheng Y., Wang Y., Zhu D. (2020). Evolution of Metallic Cardiovascular Stent Materials: A Comparative Study among Stainless Steel, Magnesium and Zinc. Biomaterials.

[19] Köster R., Vieluf D., Kiehn M., Sommerauer M., Kähler J., Baldus S., Meinertz T., Hamm C.W. (2000). Nickel and Molybdenum Contact Allergies in Patients with Coronary In-Stent Restenosis. Lancet.

[20] Kastrati A., Mehilli J., Dirschinger J., Dotzer F., Schühlen H., Neumann F.-J., Fleckenstein M., Pfafferott C., Seyfarth M., Schühlen A. (2001). Intracoronary Stenting and Angiographic Results. Circulation.

[21] Pache J.Ü., Kastrati A., Mehilli J., Schühlen H., Dotzer F., Hausleiter J.Ö., Fleckenstein M., Neumann F.-J., Sattelberger U., Schmitt C. (2003). Intracoronary Stenting and Angiographic Results: Strut Thickness Effect on Restenosis Outcome (ISAR-STEREO-2) Trial. J. Am. Coll. Cardiol..

[22] Kawamoto H., Panoulas V.F., Sato K., Miyazaki T., Naganuma T., Sticchi A., Figini F., Latib A., Chieffo A., Carlino M. (2015). Impact of Strut Width in Periprocedural Myocardial Infarction. JACC Cardiovasc. Interv..

[23] Poncin P., Proft J. Stent Tubing: Understanding the Desired Attributes. Proceedings of the Medical Device Materials: Proceedings from the Materials & Processes for Medical Devices Conference.

[24] Hermawan H., Ramdan D.P., Djuansjah J.R. (2011). Metals for Biomedical Applications. Biomedical Engineering—From Theory to Applications.

[25] Poncin P., Millet C., Chevy J., Proft J.L. Comparing and Optimizing Co-Cr Tubing for Stent Applications. Proceedings of the Medical Device Materials II: Proceedings from the Materials & Processes for Medical Devices Conference.

[26] Milleret V., Ziogas A., Buzzi S., Heuberger R., Zucker A., Ehrbar M. (2015). Effect of Oxide Layer Modification of CoCr Stent Alloys on Blood Activation and Endothelial Behavior. J. Biomed. Mater. Res. Part B Appl. Biomater..

[27] Allocco D.J., Jacoski M.V., Huibregtse B., Mickley T., Dawkins K.D. (2011). Platinum Chromium Stent Series—The TAXUS^TM^ Element^TM^ (ION^TM^), PROMUS Element^TM^ and OMEGA^TM^ Stents. Interv. Cardiol. Rev..

[28] Leone A., Simonetti F., Avvedimento M., Angellotti D., Immobile Molaro M., Franzone A., Esposito G., Piccolo R. (2022). Ultrathin Struts Drug-Eluting Stents: A State-of-the-Art Review. J. Pers. Med..

[29] (2018). Bernard Chevalier Stent Strut Thickness: Have We Reached the Minimum?. Card. Interv. Today.

[30] Park K.W., Kang S.-H., Kang H.-J., Koo B.-K., Park B.-E., Cha K.S., Rhew J.Y., Jeon H.-K., Shin E.-S., Oh J.H. (2014). A Randomized Comparison of Platinum Chromium-Based Everolimus-Eluting Stents Versus Cobalt Chromium-Based Zotarolimus-Eluting Stents in All-Comers Receiving Percutaneous Coronary Intervention. J. Am. Coll. Cardiol..

[31] Iqbal J., Gunn J., Serruys P.W. (2013). Coronary Stents: Historical Development, Current Status and Future Directions. Br. Med. Bull..

[32] Nicolas J., Pivato C.A., Chiarito M., Beerkens F., Cao D., Mehran R. (2022). Evolution of Drug-Eluting Coronary Stents: A Back-and-Forth Journey from the Bench to Bedside. Cardiovasc. Res..

[33] Schmidt T., Abbott J. (2018). Coronary Stents: History, Design, and Construction. J. Clin. Med..

[34] Aoki J., Kozuma K., Awata M., Nanasato M., Shiode N., Tanabe K., Yamaguchi J., Kusano H., Nie H., Kimura T. (2016). Three-Year Clinical Outcomes of Everolimus-Eluting Stents From the Post-Marketing Surveillance Study of Cobalt-Chromium Everolimus-Eluting Stent (XIENCE V/PROMUS) in Japan. Circ. J..

[35] Roy T., Chanda A. (2014). Computational Modelling and Analysis of Latest Commercially Available Coronary Stents During Deployment. Procedia Mater. Sci..

[36] Sangiorgi G., Melzi G., Agostoni P., Cola C., Clementi F., Romitelli P., Virmani R., Colombo A. (2007). Engineering Aspects of Stents Design and Their Translation into Clinical Practice. Ann. Ist. Super. Sanita.

[37] McQueen A., Escuer J., Schmidt A.F., Aggarwal A., Kennedy S., McCormick C., Oldroyd K., McGinty S. (2022). An Intricate Interplay between Stent Drug Dose and Release Rate Dictates Arterial Restenosis. J. Control. Release.

[38] Garg S., Serruys P.W. (2010). Coronary Stents: Current Status. J. Am. Coll. Cardiol..

[39] Hassan S., Ali M.N., Ghafoor B. (2022). Evolutionary Perspective of Drug Eluting Stents: From Thick Polymer to Polymer Free Approach. J. Cardiothorac. Surg..

[40] Garg S., Serruys P.W. (2010). Coronary Stents: Looking Forward. J. Am. Coll. Cardiol..

[41] Resolute Onyx DES Technical Specifications. https://asiapac.medtronic.com/content/dam/medtronic-com/products/coronary/stents/resolute-onyx/documents/resolute-onyx-des-technical-specifications-us.pdf?bypassIM=true.

[42] Buiten R.A., Ploumen E.H., Zocca P., Doggen C.J.M., Jessurun G.A.J., Schotborgh C.E., Roguin A., Danse P.W., Benit E., Aminian A. (2020). Thin Composite-Wire-Strut Zotarolimus-Eluting Stents Versus Ultrathin-Strut Sirolimus-Eluting Stents in BIONYX at 2 Years. JACC Cardiovasc. Interv..

[43] Bravo Baptista S. (2021). The Third Generation of Drug-Eluting Stents: Reassuring Data While We Wait for the next One. Rev. Port. Cardiol..

[44] Tittelbach M., Diener T. (2011). Orsiro—The First Hybrid Drug-Eluting Stent, Opening Up a New Class of Drug-Eluting Stents for Superior Patient Outcomes. Interv. Cardiol. Rev..

[45] Itoh T., Otake H., Kimura T., Tsukiyama Y., Kikuchi T., Okubo M., Hayashi T., Okamura T., Kuramitsu S., Morita T. (2022). A Serial Optical Frequency-Domain Imaging Study of Early and Late Vascular Responses to Bioresorbable-Polymer Sirolimus-Eluting Stents for the Treatment of Acute Myocardial Infarction and Stable Coronary Artery Disease Patients: Results of the MECHANISM-U. Cardiovasc. Interv. Ther..

[46] BioFreedom^TM^ Drug Coated Coronary Stent System Technical Specification. Biosensors Interventional Technologies Pte Ltd. https://pdf.medicalexpo.com/pdf/biosensors-international/biofreedom/75768-121795.html.

[47] CRE8TM AmphilimusTM Eluting Coronary System on Rx Balloon Catheter Alvimedica Technical Sheet. https://www.vingmed.se/wp-content/uploads/2013/10/CRE8-Technical-Data-Sheet.pdf.

[48] Serruys P.W., Onuma Y., Garg S., Vranckx P., De Bruyne B., Morice M.-C., Colombo A., Macaya C., Richardt G., Fajadet J. (2010). 5-Year Clinical Outcomes of the ARTS II (Arterial Revascularization Therapies Study II) of the Sirolimus-Eluting Stent in the Treatment of Patients With Multivessel De Novo Coronary Artery Lesions. J. Am. Coll. Cardiol..

[49] Gwon H.-C. (2018). Understanding the Coronary Bifurcation Stenting. Korean Circ. J..

[50] Nakamura S., Hall P., Maiello L., Colombo A. (1995). Techniques for Palmaz-Schatz Stent Deployment in Lesions with a Large Side Branch. Cathet. Cardiovasc. Diagn..

[51] Raphael C.E., O’Kane P.D. (2021). Contemporary Approaches to Bifurcation Stenting. JRSM Cardiovasc. Dis..

[52] Öner A., Rosam P., Borowski F., Grabow N., Siewert S., Schmidt W., Schmitz K.-P., Stiehm M. (2021). Side-Branch Expansion Capacity of Contemporary DES Platforms. Eur. J. Med. Res..

[53] Ng J., Foin N., Ang H.Y., Fam J.M., Sen S., Nijjer S., Petraco R., Di Mario C., Davies J., Wong P. (2016). Over-Expansion Capacity and Stent Design Model: An Update with Contemporary DES Platforms. Int. J. Cardiol..

[54] Gil R.J., Bil J., Kern A., Pawłowski T. (2018). First-in-Man Study of Dedicated Bifurcation Cobalt-Chromium Sirolimus-Eluting Stent BiOSS LIM C^®^—Three-Month Results. Kardiol. Pol..

[55] Kidawa M., Chiżyński K., Kacprzak M., Ledakowicz-Polak A., Zielińska M. (2017). Self-Expanding STENTYS Stents in Daily Routine Use. Kardiol. Pol..

[56] Généreux P., Kumsars I., Lesiak M., Kini A., Fontos G., Slagboom T., Ungi I., Metzger D.C., Wykrzykowska J.J., Stella P.R. (2015). A Randomized Trial of a Dedicated Bifurcation Stent Versus Provisional Stenting in the Treatment of Coronary Bifurcation Lesions. J. Am. Coll. Cardiol..

[57] Cook S., Wenaweser P., Togni M., Billinger M., Morger C., Seiler C., Vogel R., Hess O., Meier B., Windecker S. (2007). Incomplete Stent Apposition and Very Late Stent Thrombosis After Drug-Eluting Stent Implantation. Circulation.

[58] Ye Y., Qian H., Yang M., Zhu X., Gan T., Zhang S., Zeng Y. (2017). Over-Expansion of Drug-Eluting Stents in Patients with Left Main Coronary Artery Disease: An in Vivo Study. J. Int. Med. Res..

[59] Gasior P., Lu S., Ng C.K.J., Toong W.Y.D., Wong E.H.P., Foin N., Kedhi E., Wojakowski W., Ang H.Y. (2020). Comparison of Overexpansion Capabilities and Thrombogenicity at the Side Branch Ostia after Implantation of Four Different Drug Eluting Stents. Sci. Rep..

[60] Abbott Vascular XIENCE Skypoint Everolimus Eluting Coronary Stent Systems (XIENCE Skypoint EECSS). https://vascular.eifu.abbott/en/detail-screen.html.

[61] Medtronic Resolute Onyx Zotarolimus-Eluting Coronary Stent System. https://asiapac.medtronic.com/content/dam/medtronic-com/products/coronary/stents/resolute-onyx/documents/resolute-onyx-xlv-brochure-ml-2017-12.pdf.

[62] Gherbesi E., Danzi G.B. (2020). The Ultimaster Coronary Stent System: 5-Year Worldwide Experience. Future Cardiol..

[63] BALTON ALEX—Sirolimus Eluting Cobalt-Chromium Coronary Stent. https://balton.pl/images/QR_pages/Cardiovascular_catalogue.pdf.

[64] SYNERGYTM & SYNERGY MEGATRONTM EES PtCr Coronary Stent System. https://www.bostonscientific.com/en-EU/products/stents-coronary/synergy-stent-system/megatron/overexpansion.html?fbclid=IwAR30UJbHAKPLR0Gh1QgSuvtRkZ3eHwNJ3iwukbTsTVHREhxBtkZQ4XQ2CGg.

[65] Byrne R.A., Stone G.W., Ormiston J., Kastrati A. (2017). Coronary Balloon Angioplasty, Stents, and Scaffolds. Lancet.

[66] Weiss A.J., Lorente-Ros M., Correa A., Barman N., Tamis-Holland J.E. (2022). Recent Advances in Stent Technology: Do They Reduce Cardiovascular Events?. Curr. Atheroscler. Rep..

[67] Cherian A.M., Nair S.V., Maniyal V., Menon D. (2021). Surface Engineering at the Nanoscale: A Way Forward to Improve Coronary Stent Efficacy. APL Bioeng..

[68] Hong S.-J., Hong M.-K. (2022). Drug-Eluting Stents for the Treatment of Coronary Artery Disease: A Review of Recent Advances. Expert Opin. Drug Deliv..

[69] Scoutaris N., Ross S., Douroumis D. (2016). Current trends on medical and pharmaceutical applications of inkjet printing technology. Pharm Res..

[70] Scoutaris N., Chai F., Maurel B., Sobocinski J., Zhao M., Moffat J.G., Craig D.Q., Martel B., Blanchemain N., Douroumis D. (2016). Development and biological evaluation of inkjet printed drug coatings on intravascular stent. Mol Pharm..

[71] Polimeni A., Sorrentino S., Spaccarotella C., Mongiardo A., Sabatino J., De Rosa S., Gori T., Indolfi C. (2020). Stent Thrombosis After Percutaneous Coronary Intervention. Cardiol. Clin..

[72] Gil R.J., Bil J., Legutko J., Pawłowski T., Gil K.E., Dudek D., Costa R.A. (2018). Comparative Assessment of Three Drug Eluting Stents with Different Platforms but with the Same Biodegradable Polymer and the Drug Based on Quantitative Coronary Angiography and Optical Coherence Tomography at 12-Month Follow-Up. Int. J. Cardiovasc. Imaging.

[73] Lhermusier T., Ohayon P., Boudou N., Bouisset F., Campelo-Parada F., Roncalli J., Elbaz M., Carrié D. (2019). Re-Endothelialisation after Synergy Stent and Absorb Bioresorbable Vascular Scaffold Implantation in Acute Myocardial Infarction: COVER-AMI Study. Trials.

[74] Suwannasom P., Athiksakul S., Thonghong T., Lertsuwunseri V., Chaipromprasit J., Srimahachota S., Udayachalerm W., Kuanprasert S., Buddhari W. (2021). Clinical Outcomes of an Ultrathin-Strut Sirolimus-Eluting Stent in All-Comers Population: Thailand Orsiro Registry. BMC Cardiovasc. Disord..

[75] Chisari A., Pistritto A., Piccolo R., La Manna A., Danzi G. (2016). The Ultimaster Biodegradable-Polymer Sirolimus-Eluting Stent: An Updated Review of Clinical Evidence. Int. J. Mol. Sci..

[76] Menown I.B.A., Mamas M.A., Cotton J.M., Hildick-Smith D., Eberli F.R., Leibundgut G., Tresukosol D., Macaya C., Copt S., Slama S.S. (2021). Thin Strut CoCr Biodegradable Polymer Biolimus A9-Eluting Stents versus Thicker Strut Stainless Steel Biodegradable Polymer Biolimus A9-Eluting Stents: Two-Year Clinical Outcomes. J. Interv. Cardiol..

[77] Todd Neale New-Generation DES Better than Older Stents Over 10 Years, Regardless of Polymer Type. https://www.tctmd.com/news/new-generation-des-better-older-stents-over-10-years-regardless-polymer-type.

[78] de Abreu-Silva E.O., Costa R.A., Abizaid A., Ramondo A., Brenot P., Benamer H., Desideri A., Berland J., Almeida B.O., Digne F. (2012). Long-Term Clinical and Angiographic Follow-up of the New Non-Polymeric Paclitaxel-Eluting Stent for the Treatment of De Novo Coronary Lesions: Outcomes of the PAX-B Study. Rev. Bras. Cardiol. Invasiva.

[79] Tan S., Nogic J., Thein P., Nerlekar N., Cameron J., Nasis A., West N., Brown A. (2018). TCTAP A-100 Polymer-Free Versus Biodegradable Polymer Drug-Eluting Stents for the Treatment of Coronary Artery Disease: A Meta-Analysis of Randomized Trials. J. Am. Coll. Cardiol..

[80] Nogic J., Thein P., Mirzaee S., Comella A., Soon K., Cameron J.D., West N.E.J., Brown A.J. (2019). Biodegradable-Polymer Versus Polymer-Free Drug-Eluting Stents for the Treatment of Coronary Artery Disease. Cardiovasc. Revasc. Med..

[81] Gao K., Sun Y., Yang M., Han L., Chen L., Hu W., Chen P., Li X. (2017). Efficacy and Safety of Polymer-Free Stent versus Polymer-Permanent Drug-Eluting Stent in Patients with Acute Coronary Syndrome: A Meta-Analysis of Randomized Control Trials. BMC Cardiovasc. Disord..

[82] Wu J.J., Way J.A.H., Kritharides L., Brieger D. (2019). Polymer-Free versus Durable Polymer Drug-Eluting Stents in Patients with Coronary Artery Disease: A Meta-Analysis. Ann. Med. Surg..

[83] Chen Y., Zeng Y., Zhu X., Miao L., Liang X., Duan J., Li H., Tian X., Pang L., Wei Y. (2021). Significant Difference between Sirolimus and Paclitaxel Nanoparticles in Anti-Proliferation Effect in Normoxia and Hypoxia: The Basis of Better Selection of Atherosclerosis Treatment. Bioact. Mater..

[84] Bangalore S., Kumar S., Fusaro M., Amoroso N., Attubato M.J., Feit F., Bhatt D.L., Slater J. (2012). Short- and Long-Term Outcomes With Drug-Eluting and Bare-Metal Coronary Stents. Circulation.

[85] Schömig A., Dibra A., Windecker S., Mehilli J., Suárez de Lezo J., Kaiser C., Park S.-J., Goy J.-J., Lee J.-H., Di Lorenzo E. (2007). A Meta-Analysis of 16 Randomized Trials of Sirolimus-Eluting Stents Versus Paclitaxel-Eluting Stents in Patients With Coronary Artery Disease. J. Am. Coll. Cardiol..

[86] Stettler C., Wandel S., Allemann S., Kastrati A., Morice M.C., Schömig A., Pfisterer M.E., Stone G.W., Leon M.B., de Lezo J.S. (2007). Outcomes Associated with Drug-Eluting and Bare-Metal Stents: A Collaborative Network Meta-Analysis. Lancet.

[87] Ostojic M., Sagic D., Jung R., Zhang Y.-L., Nedeljkovic M., Mangovski L., Stojkovic S., Debeljacki D., Colic M., Beleslin B. (2008). The Pharmacokinetics of Biolimus A9 after Elution from the Nobori Stent in Patients with Coronary Artery Disease: The NOBORI PK Study. Catheter. Cardiovasc. Interv..

[88] Oh S., Hyun D.Y., Cho K.H., Kim J.H., Jeong M.H. (2021). Comparison of Long-Term Clinical Outcomes among Zotarolimus-, Everolimus-, and Biolimus-Eluting Stents in Acute Myocardial Infarction Patients with Renal Impairment. Cardiol. J..

[89] Tada T., Byrne R.A., Cassese S., King L., Schulz S., Mehilli J., Schömig A., Kastrati A. (2013). Comparative Efficacy of 2 Zotarolimus-Eluting Stent Generations: Resolute versus Endeavor Stents in Patients with Coronary Artery Disease. Am. Heart J..

[90] Bozsak F., Gonzalez-Rodriguez D., Sternberger Z., Belitz P., Bewley T., Chomaz J.-M., Barakat A.I. (2015). Optimization of Drug Delivery by Drug-Eluting Stents. PLoS ONE.

[91] Xu B., Gao R., Yang Y., Cao X., Qin L., Li Y., Li Z., Li X., Lin H., Guo Y. (2016). Biodegradable Polymer-Based Sirolimus-Eluting Stents With Differing Elution and Absorption Kinetics. J. Am. Coll. Cardiol..

[92] Habib A., Finn A.V. (2016). Antiproliferative Drugs for Restenosis Prevention. Interv. Cardiol. Clin..

[93] Valgimigli M., Frigoli E., Heg D., Tijssen J., Jüni P., Vranckx P., Ozaki Y., Morice M.-C., Chevalier B., Onuma Y. (2021). Dual Antiplatelet Therapy after PCI in Patients at High Bleeding Risk. N. Engl. J. Med..

